# Editorial: Epigenetic regulation and non-histone post-translational modification in cancer

**DOI:** 10.3389/fgene.2023.1176174

**Published:** 2023-04-06

**Authors:** Jiang Luo, Zhengrong Huang, Wei Wei, Yingming Sun, Yan Gong

**Affiliations:** ^1^ Department of Biological Repositories, Tumor Precision Diagnosis and Treatment Technology and Translational Medicine, Hubei Engineering Research Center, Zhongnan Hospital of Wuhan University, Wuhan, China; ^2^ Brain Research Center, Department of Neurosurgery, Zhongnan Hospital of Wuhan University, Wuhan, China; ^3^ Department of Radiation and Medical Oncology, Affiliated Sanming First Hospital of Fujian Medical University, Sanming, China

**Keywords:** epigenetic regulation, non-histone post-translational modification, cancer, DNA methylation, m6A modifications, non-coding RNAs

Epigenetic changes are essentially involved in both normal organism function and disease progression (Deans and Maggert, 2015). Epigenetic regulation includes DNA methylation or demethylation, chromatin remodeling, histone modifications and non-coding RNAs, which are broadly reported to dysfunction in cancer. Of noted, it is increasingly clear that epigenetic regulation parallels with gene expression modulation. Currently, significant progress has been made in the development of drugs targeting key enzymes involved in epigenetic regulation and post-translational modification without histone. Several drugs have been approved for therapeutic application, and many more are in clinical and preclinical testing (Ferreira and Esteller, 2018; Lu et al., 2020). The aim of this Research Topic is to provide an overview of the current understanding and fundamental findings in the field of epigenetic regulation and non-histone post-translational modifications in cancer. We collected 14 articles including the effects of m6A modifications, non-coding RNAs and SELENBP1 in cancer progression.

DNA methylation is strongly associated with cancer, and hypermethylation of some genes in the promoter region interferes with the reading of DNA information thereby altering epigenetics, thus it has the potential to be a promising target for cancer therapy (Smith and Meissner, 2013). Liexi Xu et al. used the methylation and clinical data of lung adenocarcinoma (LUAD) patients from TCGA. They found 11 differential methylation genes and established a methylation scoring model to assess prognosis, suggesting that these genes could be used as biomarkers of methylation in LUAD. Dong-Mei Hu et al. focused on the relationship between Forkhead box P (FOXP) family DNA methylation and immune-related factors in non-small cell lung cancer (NSCLC) patients. FOXP family is widely involved in regulating immune molecules and influencing immune infiltration in NSCLC, and FOXP family DNA methylation is associated with NSCLC prognosis. In addition to lung cancer, immunosuppression and immune cells dysfunction are critical to the development of colonic adenocarcinoma (COAD) (Gatenbee et al., 2022). Salem Baldi et al. analyzed the relationship between ARID1B expression, DNA methylation and prognosis in COAD patients based on TCGA, and found that differences in immune cell infiltration were associated with ARID1B expression, and that ARID1B was hypermethylated in COAD tissues. Since the ARID1B methylation levels were negatively associated with mRNA levels, low ARID1B expression was an important indicator of poor prognosis, and ARID1B hypermethylation could be an early diagnostic biomarker in COAD. Furthermore, Xingyu Liu et al. obtained 4 alcohol-related cancer samples from TCGA and GEO databases, and identified a total of 193 differentially methylated probes. By enrichment analysis of differential genes, they concluded that the alcohol might facilitate transcriptional dysfunction *via* inducing the methylation status of transcriptional regulators, leading to tumor development. They also identified the hypermethylated CpG island (chr19:58220189-58220517), which regulated the transcriptional activity of zinc-finger protein 154 as a potential therapeutic biomarker.

In addition to DNA modifications, RNA modifications are prevalent in mammalian cells, and provide a new dimension to regulate gene expression. Among them, N6-methyladenosine (m6A) modifications are the most common in eukaryotic mRNA. Shaojie Li et al. obtained sample data of head and neck squamous cell carcinoma (HNSCC) from TCGA and GEO databases. Via analyzing the correlation between m6A regulator expression and immune scores, they found that the HNSCC patients could be divided into 2 groups based on m6A reader genes (IGF2BP2 and YTHDF1). Low YTHDF1 and IGF2BP2 expressing patients have more immune cells enriched in TME and better prognosis. Mengying Zhou et al. reviewed the role of different m6A-related enzymes in breast cancer, and concluded that m6A-related genes could be used as not only markers for diagnosis and prognosis prediction, but also effective targets for breast cancer treatment.

Moreover, non-coding RNA transcriptional modifications are also crucial (Yao et al., 2022). With the continuous researches on long non-coding RNA (lncRNA), it was found that lncRNA play important roles in various biological regulatory processes. Jing Huang et al. downloaded mRNA expression data from the TCGA database of HNSCC patients, identified 1,117 lncRNAs associated with necrosis, of which 55 ones were associated with patient survival. They selected 24 genes that positively regulated necroptosis to establish a new risk scoring model for assessing HNSCC patient prognosis. Jing Hu et al. obtained information about autophagy-related genes in NSCLC patients from TCGA and HADb, and identified 7 autophagy-related lncRNAs whose composition of risk models could accurately predict the prognosis of NSCLC patients. ABALON deficiency in A549 and NCI-H292 significantly inhibited the proliferation and metastasis of NSCLC cells and promoted autophagy. Wei Yu et al. identified 298 lncRNAs associated with cuproptosis using TCGA data of gastric adenocarcinoma patients, including 13 lncRNAs associated with survival, and further identified 9 lncRNAs by LASSO regression method. Based on these 9 lncRNAs, they established a risk assessment model to evaluate the prognosis and sensitivity of patients to therapeutic drugs.

Immunotherapy combined with radiotherapy is one of the best combinations for oncotherapy. Increasing researches suggested that lncRNA might be associated with the responses to immunotherapy and radiotherapy. Chuanhao Zhang et al. acquired RNA-seq data and clinical characteristics of 594 LUAD patients from TCGA, identified 2,093 N7-methylguanosine related lncRNAs, and finally constructed a risk-prognosis model *via* screening 6 prognosis-related lncRNAs, which not only accurately predicted the patient survival, but also reflected the immune characteristics of LUAD patients and provided better guidance for individualized patient treatment. Jianqing Zheng et al. screened 26 immune-related lncRNAs (ir-lncRNAs) differentially expressed in radiation-resistant esophageal squamous cell carcinoma by the GEO database and paired the differentially expressed lncRNA with each other in the GSE45670 dataset to construct 325 ir-lncRNA pairs, they established a prognostic risk model based on 3 pairs of ir-lncRNA, and suggested that macrophage infiltration and differential expression of ir-lncRNA are potential mechanisms of resistance to radiotherapy. Moreover, Linghui Jia et al. collected 58 patients with oral squamous cell carcinoma, and found that the expression of CircPUM1 (a circular RNA) was significantly increased in oral squamous cell carcinoma. CircPUM1 downregulation induced mir-580, which inhibited STAT3 expression, induced apoptosis and enhanced radiosensitivity.

Histones, as essential components of nucleosomes, play an important role in the structure of chromosomes. Histone modifications are considered important epigenetic mechanisms for gene expression regulation, and small molecule inhibitors have been developed to detect the effects of these modifications on cellular proteins (Buuh et al., 2018). SETD2 is the major methyltransferase catalyzing histone H3K36. Zihang Zeng et al. collected data on LUAD patients from GEO and TCGA, and through multi-omics analysis identified that SETD2 was associated with radiosensitivity. SETD2 downregulation attenuated proliferation and migration, and enhanced the apoptosis and radiosensitivity of LUAD cells. They also found that reducing m6A-related genes (RBM2 or YTHDF15) could enhance the protective effect of SETD2 on patient prognosis. Yue Zhang et al. reviewed the function and regulatory mechanisms of SELENBP1 (a selenium-binding protein) during cancer progression, and also discussed potential cancer treatment strategies targeting SELENBP1 epigenetic modifications.

Cancer is not only a genetic disease, but also an epigenetic disease. Epigenetic mechanisms are engaged in the regulation of many aspects of cancer biology (Garcia-Martinez et al., 2021). With the development of mass spectrometry-based proteomics technologies, some non-histone modifications (e.g., lysine acetylation, lactylation) also play key roles in cell growth, metabolism, and signal transduction (Narita et al., 2019; Yang et al., 2023). This Research Topic focused on the regulation of epigenetic and non-histone post-translational modifications, as well as their impacts on cancer development and progression. Patterns of epigenetic regulation and non-histone modifications may be potential predictors of cancer patient prognosis and survival, providing novel insights into the oncotherapy. We hope more researches devote to this field in the future and look forward to their early translation into clinical treatments.
